# Supplementary material for: Switchable genome editing via genetic code expansion
Source: Sci Rep. 2018 Jul 3;8:10051. doi: 10.1038/s41598-018-28178-3 (PMC6030211; doi:10.1038/s41598-018-28178-3)
Supplement: Supplementary file 1 — Supplementary information [file 41598_2018_28178_MOESM1_ESM.pdf]

Supplementary information

Switchable genome editing *via* genetic code expansion

Toru Suzuki[1], Maki Asami[1], Sanjay G. Patel[2], Louis Y. P. Luk[2], Yu-Hsuan Tsai[2] and Anthony C. F. Perry[1]

[1]Laboratory of Mammalian Molecular Embryology, Department of Biology and Biochemistry, University of Bath, BA2 7AY, England. [2]School of Chemistry, Cardiff University, Main Building, Park Place, Cardiff, CF10 3AT, Wales.

Supplementary figure legends

Supplementary Figure S1. Strategy to expand the genetic code for BOC-induced eGFP^{N150B} expression from transgene DNA injected into mouse oocytes. (**A**) Structure of the transgene (tg) construct (*eGFP^{N150B}* tg) used in the experiments of (**B**) to (**D**). (**B**) Schematic showing how the *eGFP^{N150B}* tg cassette is injected with sperm into wild-type metaphase II (mII) oocytes. Sperm injection fertilizes the oocyte and subsequent culture is in the presence or absence of BOC, as indicated. (**C**) Vertically paired images showing day 4 (E4.0) embryos generated per the method depicted in (**B**) by injecting B6D2F1 sperm into B6D2F1 mII oocytes, with culture in the absence of BOC or with 10 mM BOC added in the final 24 h of culture. Bright field (BF) is shown (upper) with eGFP (fluorescence). Scale bar, 100 μm. (**D**) Histograms depicting percentage of 1-cell embryos developing to the blastocyst stage (open bars) and of green fluorescent embryos (green bars) on embryonic day 4 (E4.0) in the experiment of (**B**) and (**C**). Numbers of embryos (*n*) are shown above corresponding columns.

Supplementary Figure S2. Expanding the genetic code for BOC-induced eGFP^{N150B} expression from cRNA injected into mouse oocytes. (**A**) Schematic depicting injection of a metaphase II (mII) oocyte with the RNAs indicated and culture in the absence or presence of BOC for 5 h or 24 h. In these experiments, oocytes were visualized 5 and 24 h after injection. (**B**) Histograms showing (±s.e.m.) fluorescence levels in arbitrary units (a.u.) of oocytes injected per the protocol of (**A**) and exposed to BOC at the concentrations indicated for 5 h. Concentrations of injected RNAs were: *eGFP^{N150B}* cRNA (600 ng/μl), *Pyl* tRNA (1,200 ng/μl) and *PylRS* cRNA (600 ng/μl). (**C**) As per (**B**) except that oocytes were exposed for the entire time-course of 24 h to BOC at the concentrations indicated. (**D**) Vertically paired bright field (BF) and fluorescence images with broad wavelength U.V. illumination of oocytes of (**C**)

after injection with RNAs indicated below and incubation for 24 h in BOC at the concentrations indicated. Bar, 100 μm.

Supplementary Figure S3. Delineation of BOC-induced Cas9^{K510B} and Cas9^{K742B} activities in human embryonic kidney fibroblasts cultured *in vitro*. (**A**) Paired micrographs showing representative HEK293 cells following co-transfection with an expression construct encoding the HA-tagged Cas9 mutant indicated, plus another construct encoding GFP, PylRS, gRNA and *Pyl* tRNA. Transfectants were incubated with (+) or without BOC (1 mM) for 24 h prior to image capture. In these experiments, loss of the GFP signal indicates Cas9 activity. Scale bar, 20 μm. (**B**) Immunoblot (IB) analysis of cells of (**A**) probed with antibodies against HA (Cas9) or GFP (including a longer exposure, long x), showing the full-length filter of Figure 2A. Uniform gel loading and membrane transfer were respectively confirmed with the Bio-Rad stain-free system (BR) and Ponceau S (PS). m, markers. All panels are of the same representative blot.

Supplementary Figure S4. BOC-induced edits in embryos and offspring. (**A**) Genomic sequences of the edited region of *eGFP* in the non-fluorescent offspring of Figure 4A. Sequences of two randomly-selected fluorescent pups were identical to the corresponding region of wild-type *eGFP*. (**B**) Genomic sequences of the targeted region of *Sry* in phenotypic females of Figure 5A, produced by BOC-induced editing. Labeling for is as per (**A**). (**C**) Genomic sequences of part of *Tyr* in offspring with altered coat color phenotypes following BOC-induced editing. Labeling for is as per (**A**). (**D**) BOC-induced editing revealed by sequence analysis of cDNA derived from individual non-fluorescing blastocysts of Figure 5C. In brief, mII oocytes from *pZP3-Cas9*^{K510B} transgenic females were injected with *Pyl* tRNA (1,200 ng/μl), *eGFP* gRNA (200 ng/μl) and *PylRS* cRNA (600 ng/μl) and incubated in the

presence of 1 mM BOC for 4-5 h, followed by injection of an *eGFP*$^{+}$ sperm and embryo culture to the blastocyst stage (E4.5). Non-fluorescent blastocysts all contained edits (*n*=4) whereas control, fluorescent blastocysts when BOC was omitted did not.

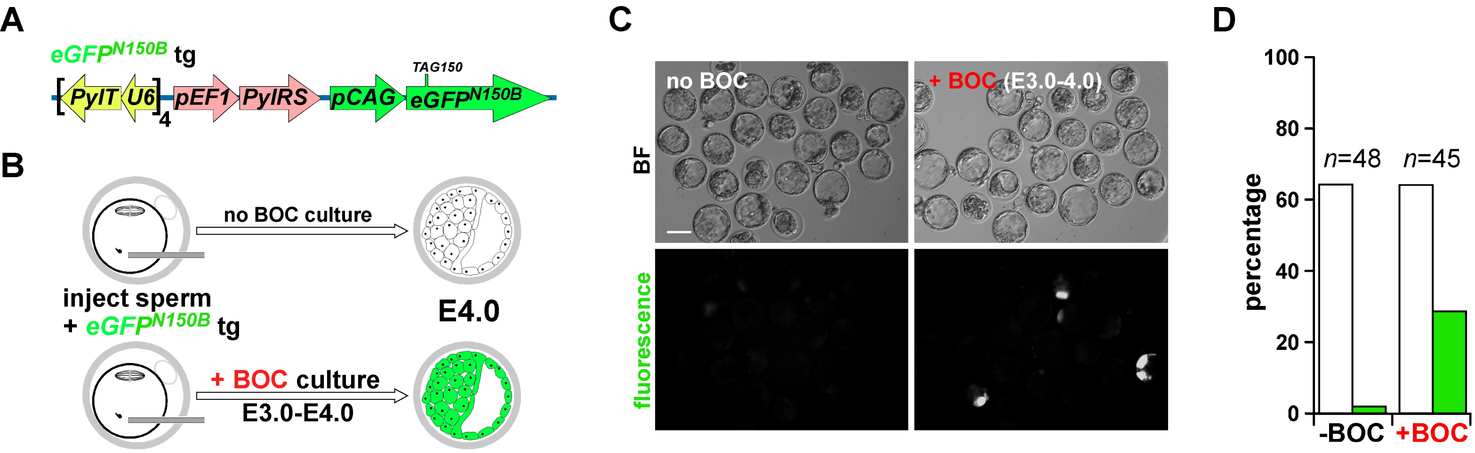

Supplementary Figure S1
Suzuki *et al.*

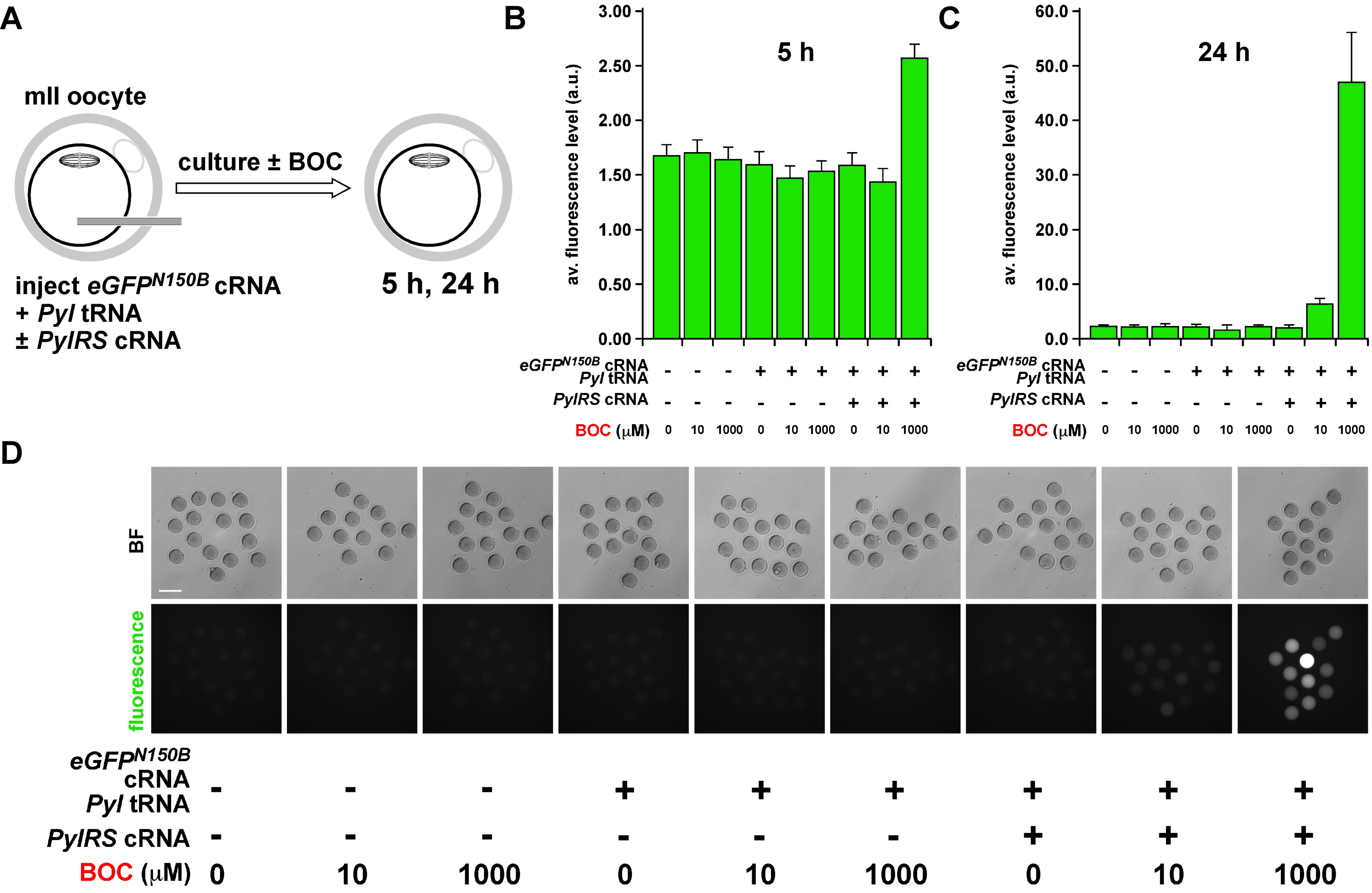

Supplementary Figure S2

Suzuki *et al.*

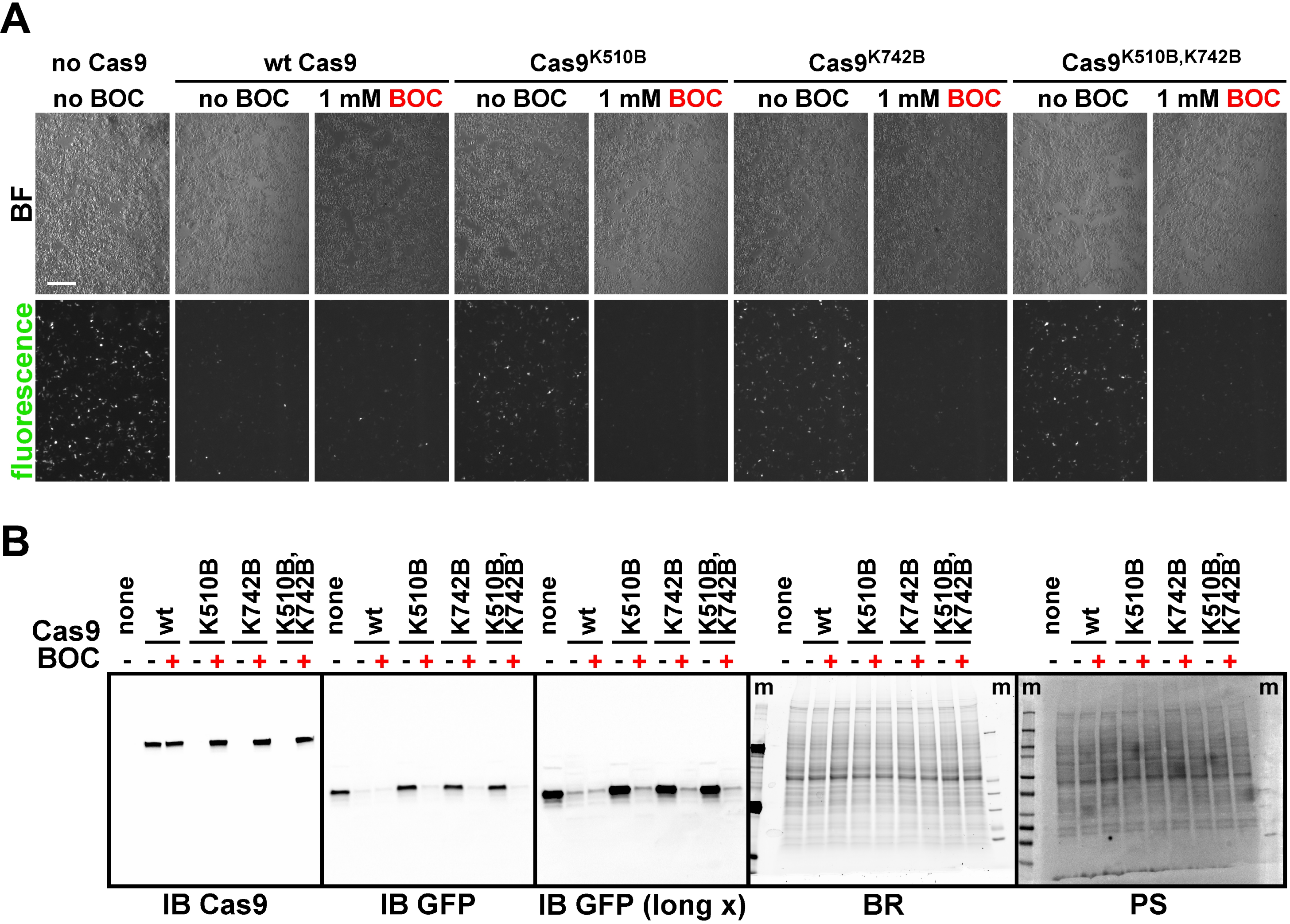

Supplementary Figure S3
Suzuki *et al*.

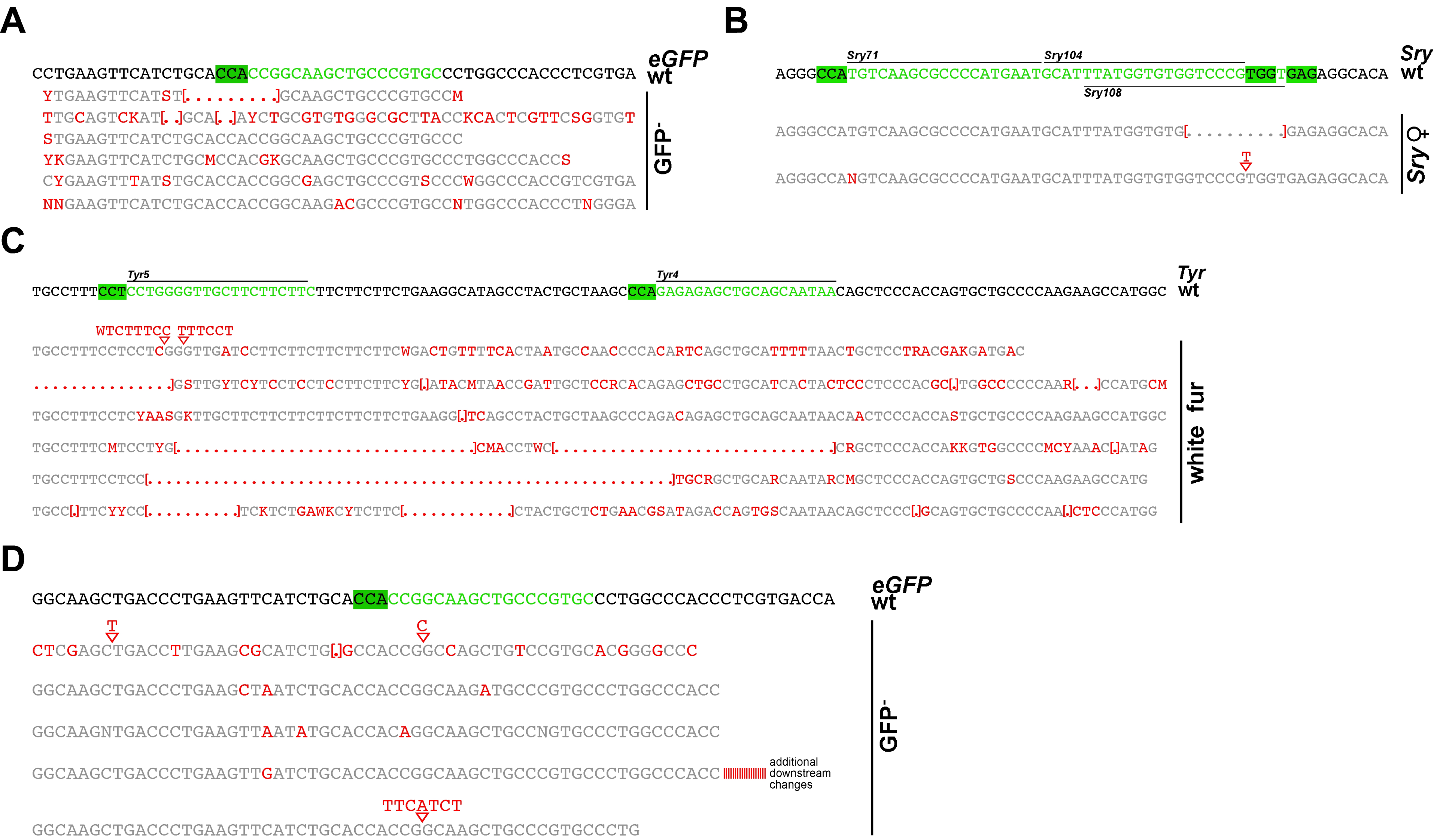

Supplementary Figure S4
Suzuki *et al.*

Supplementary Table S1. PCR primers used in this work

designation	sequence (5'→3')
eGFP S	GACGTAAACGGCCACAAGTT
eGFP AS	GTCCTCCTTGAAGTCGATGC
SryF	AAGCTTTGCTGGTTTTTGGA
SryR	GCAGGTGGAAAAGCCTTACA
Sry22	CAAGTTTTGGGACTGGTGACA
Sry465	CAACAGGCTGCCAATAAAAGC
F Tyr g3 317	AATAGGACCTGCCAGTGCTCA
R Tyr g3 939	GGTGTTCCATCGCATAAAACC
Tyr2F	CTTCCTAGGGAGGAAAGGGTGCTTGAG
Tyr2R	AGGCAGAGCATTCCCATCTCTTGCCTG
gRNA *Sry71*	GATCACTAATACGACTCACTATAGGTAAGTACCCCGCGAACTGTGT<br>TTTAGAGCTAGAAAT
gRNA *Sry104*	GATCACTAATACGACTCACTATAGGGCATTTATGGTGTGGTCCCG<br>GTTTTAGAGCTAGAAAT
gRNA *Sry108*	GATCACTAATACGACTCACTATAGGTTATGGTGTGGTCCCGTGGT<br>GTTTTAGAGCTAGAAAT
gRNA *Tyr3*	GATCACTAATACGACTCACTATAGGGTGTCAAGGGACACACTGCT<br>GTTTTAGAGCTAGAAAT
gRNA *Tyr4*	GATCACTAATACGACTCACTATAGGGTTATTGCTGCAGCTCTCTCG<br>TTTTAGAGCTAGAAAT
gRNA *Tyr5*	GATCACTAATACGACTCACTATAGGGAAGAAGAAGCAACCCCAGG<br>GTTTTAGAGCTAGAAAT
gRNA *eGFP1*	GATCACTAATACGACTCACTATAGGGCACGGGCAGCTTGCCGGGT<br>TTTAGAGCTAGAAAT
F1	GTCTATATCATGGCCGACAAGCAG
R1	TTGTCGGCCATGATATAGACCTAGTGGCTGTTGTAGTTGTACTCC
F2	CACCAACCTGTGATGATAAGGATCCACTAGTCCAGTGTGG
R2	TCACCATTCCGGACGCCATGGTTG
F3	CATGGCGTCCGGAATGGTGAGCAAGGGCGAGGAG
R3	TGTAATCCAGAGGTTGATTGTCGACTCACTTGTACAGCTCGTCCAT<br>GC
F4	CACCAACCTGTGATGATAAGGATCCACTAGTCCAGTGTGGTGGAA<br>TTGACATTGATTATTGACTAGTTATTAATAGTAATCAATTACGGGGT<br>CATTAG
R4	TGTAATCCAGAGGTTGATTGTCGACCCCTCCCATATGTCCTTCCGA<br>GTG
F5	CAGCATCGGCCTGGACATCGGTACC
R5	TTCACCTTGGTCAGCTCGTTGTACACGGTGAAGTACTCGTACAGC<br>AGGCTGTGCTAGGGCAGCACCTTCTCG
F6	CTGTACGAGTACTTCACCGTGTACAACG
R6	TCCACCACCTACACGGTCTGCAGGATGC

F7	CAGACCGTGTAGGTGGTGGACGAGCTG
R7	CTGTCCAGGATCTGGGCCACGTG
F12	CCGGCAAGCTGCCCGTGCGTTTTAGAGCTAGAAATAGCAAGTTAAAATAAGG
R12	ACGCACGGGCAGCTTGCCGGCGGTGTTTCGTCCTTTCCACAAG
F13	CGGGCACCGGAGCGATCGCAGATCTGGGCAGGAAGAGGGCCTATTTC
R13	GCCCTCTTCCTGCCCAACTAGAATTCAAAAAAAGCACCGACTCGGTGCC
S1	GTAAACGGCCACAAGTTC
S2	TAGCGGCTGAAGCACTG
S4	GCTGCACCAGAGAGAAC
S5	ACGTGGACAAGCTGTTC
S6	ACAGGCCGTTCTTCTTC
S7	AGCCCATCCTGGAGAAG
S8	GACAGCGTGGAGATCAG
S9	GCAGCCAGATCCTGAAG
S16	GCCTATTTCCCATGATTCC
S17	ACTGCAAACTACCCAAG
S18	AGCCAGTACACGACATC
pZP3 943 F	AGACTTCCCAGAGTTGGTTG
pZP3 943 R	GTGACATCAGCTTCATCGGT

Target sequences in primers used for gRNA expression vector construction are underlined.
